# Persistence, use of resources and costs in patients under migraine preventive treatment: the PERSEC study

**DOI:** 10.1186/s10194-022-01448-2

**Published:** 2022-07-07

**Authors:** Pablo Irimia, David García-Azorín, Mercedes Núñez, Sílvia Díaz-Cerezo, Pepa García de Polavieja, Tommaso Panni, Aram Sicras-Navarro, Antoni Sicras-Mainar, Antonio Ciudad

**Affiliations:** 1 Department of Neurology, Headache Unit, University Clinic of Navarra, Av. Pio XII 36, 31008 Pamplona, Spain; 2 Headache Unit, Department of Neurology, Hospital Clínico Universitario de Valladolid, Av. Ramón y Cajal 3, 47003 Valladolid, Spain; 3 Lilly Spain, Av. de la Industria 30, 28108 Madrid, Spain; 4 Lilly Deutschland GmbH, Werner-Reimers-Strasse 2-4, 61352 Bad Homburg, Germany; 5 Atrys Health, HEOR, 392 PB, 08025 Barcelona, Carrer Provença Spain

**Keywords:** Migraine disorders, Healthcare costs, Health resources, Therapeutics, Pain management, Prevention and control, Proportional hazards models, Retrospective studies, Spain

## Abstract

**Background:**

Migraine represents a serious burden for national health systems. However, preventive treatment is not optimally applied to reduce the severity and frequency of headache attacks and the related expenses. Our aim was to assess the persistence to traditional migraine prophylaxis available in Spain and its relationship with the healthcare resource use (HRU) and costs.

**Methods:**

Retrospective observational study with retrospective cohort design of individuals with migraine treated with oral preventive medication for the first time from 01/01/2016 to 30/06/2018. One-year follow-up information was retrieved from the Big-Pac™ database. According to their one-year persistence to oral prophylaxis, two study groups were created and describe regarding HRU and healthcare direct and indirect costs using 95% confidence intervals (CI). The analysis of covariance (ANCOVA) was performed as a sensitivity analysis. Patients were considered persistent if they continued on preventive treatment until the end of the study or switched medications within 60 days or less since the last prescription. Non-persistent were those who permanently discontinued or re-initiated a treatment after 60 days.

**Results:**

Seven thousand eight hundred sixty-six patients started preventive treatment (mean age (SD) 48.2 (14.8) and 80.4% women), of whom 2,545 (32.4%) were persistent for 6 months and 2,390 (30.4%) for 12 months. Most used first-line preventive treatments were antidepressants (3,642; 46.3%) followed by antiepileptics (1,738; 22.1%) and beta-blockers (1,399; 17.8%). The acute treatments prescribed concomitantly with preventives were NSAIDs (4,530; 57.6%), followed by triptans (2,217; 28.2%). First-time preventive treatment prescribers were mostly primary care physicians (6,044; 76.8%) followed by neurologists (1,221; 15.5%). Non-persistent patients required a higher number of primary care visits (mean difference (95%CI): 3.0 (2.6;3.4)) and days of sick leave (2.7 (0.8;4.5)) than the persistent ones. The mean annual expenditure was €622 (415; 829) higher in patients who not persisted on migraine prophylactic treatment.

**Conclusions:**

In this study, we observed a high discontinuation rate for migraine prophylaxis which is related to an increase in HRU and costs for non-persistent patients. These results suggest that the treatment adherence implies not only a clinical benefit but also a reduction in HRU and costs.

**Supplementary Information:**

The online version contains supplementary material available at 10.1186/s10194-022-01448-2.

## Background

Migraine is a complex neurological disease characterized by recurrent moderate-to-severe headache attacks that usually last hours to a few days [1, 2]. According to the most recent Global Burden of Disease study, migraine affects approximately one billion people across all countries [3, 4]. In 2019, it was responsible for 42.1 million years lived with disability (YLDs), which represented a 4.8% of total YLDs [4], and it was ranked the second leading cause of disability in all ages, especially in young women aged 15 to 49 years [5]. In Spain, over five million people present with migraine, rising the one-year prevalence up to 12–13% [6, 7]. Of them, 80% are women who are most affected in their most productive years [6, 7].

Due to its high prevalence, disability burden and consequences on other domains of life (social, family, career, etc.), migraine poses a significant public health issue [8]. From the economic standpoint, migraine total expenditure in Europe was calculated at €50–€111 billion, which incurred through direct (7%) and indirect (93%) healthcare costs [9]. Absenteeism and productivity losses account for most indirect costs, whereas direct costs are related to medication and healthcare resource use (HRU) (i.e. outpatient, inpatient and emergency service) to manage patients with migraine and their treatment-related side effects [9–13].

The therapeutic approach for people with migraine is based on the avoidance or modification of triggers and the concomitant administration of pharmacological and adjunct non-pharmacological treatments for acute symptom relief and/or prophylaxis [2]. Most treatment guidelines concur on the use of oral preventive treatments as first and second line therapies. These drugs belong to a wide variety of therapeutic families, having different mechanisms of action and distinct tolerability profiles: beta-blockers, antidepressants, antiepileptics/neuromodulators, calcium antagonists, angiotensin-converting enzyme (ACE) inhibitors and angiotensin II receptor blockers (ARBs), as well as injectable botulinum toxin (in chronic migraine) [2, 11, 14, 15]. Recently, more specific molecules have been developed for the acute (gepants and ditans) and prophylactic (monoclonal antibodies to calcitonin gene-related peptide (CGRP) and gepants) management of these patients. Nevertheless, their high cost and restricted availability currently limit their use [2, 14].

The goals of preventive treatment are to reduce the attack frequency, severity, duration and migraine-related disability; to improve the responsiveness to acute treatment and to reduce the economic impact of the disease [15]. Nevertheless, preventives are not exempt from limitations. None of the traditional preventive drugs available in Spain before 2019 has been specifically designed for migraine, the efficacy is moderate and most treatments present safety and tolerability issues [15]. This may explain, at least in part, the low proportion of patients under optimal prophylaxis [9, 16–19]. According to a recent survey, Spain showed the highest rate of adequately treated patients in contrast to other European countries, but still this was as low as 13.7% [20].

Many authors have suggested that improving persistence to preventive medication offers an opportunity to diminish headache-related disability, prevent migraine progression and improve patient outcomes [17, 21, 22]. Successful migraine prophylaxis may also contribute to the well-functioning of healthcare systems, decreasing the direct and indirect costs of the disease, however, few studies have specifically addressed this matter in the context of migraine. In a recent quantitative analysis, authors found that HRU related to treatment discontinuation across four European countries was high. More than 80% of patients who failed at least two preventive treatments required one or more outpatient visits for migraine, 27% attended the accident & emergency department (A&E) and 5% had to be hospitalized [10].

The main objectives of this study were to describe the persistence and prescription patterns of available treatments for migraine prevention in real-world settings as well as the relationship between persistence and HRU together with the associated costs. Additionally, we aim to explore the associated factors for preventive treatment discontinuation.

## Methods

### Study design and participants

The PERSEC study was an observational retrospective cohort study based on electronic medical records in the Big-Pac™ database (Atrys Health-Real Life Data, Madrid, Spain) [23], which collects and unifies computerized and anonymized patient medical records from primary and secondary care, records of drug dispensation and other complementary databases from seven autonomous regions of Spain. The Big-Pac™ database is registered with The European Network of Centres for Pharmacoepidemiology and Pharmacovigilance (ENCePP), a network coordinated by the European Medicines Agency. The population assigned to the health centres from which data were extracted included 1,832,356 inhabitants at the time of data extraction and could be considered representative of the Spanish population [23].

The study population were adult patients with migraine who were prescribed preventive medication (index date) between 01/01/2016 and 30/06/2018. One-year follow-up (until 30/06/2019) and one-year preindex (from 01/01/2015 to 31/12/2015) periods were added to extract the variables needed to cover the study objectives.

### Eligibility criteria

Inclusion criteria were:

1. Age ≥ 18 years at the index date.
2. Confirmed diagnosis of migraine as per the International Classification of Diseases (10^th^ edition) Clinical Modification (ICD-10-CM; Code G43) before the index period.
3. First prescription of migraine prophylactic medication, including beta-blockers, antidepressants, antiepileptics/neuromodulators, calcium antagonists, angiotensin-converting enzyme (ACE) inhibitors or angiotensin II receptor blockers (ARBs) which is linked to the corresponding ICD-10-CM; Code G43.
4. Healthcare assistance required during the index period.

Exclusion criteria were:

1. Prescription of the aforementioned medications before the diagnosis of migraine, i.e. for any indication other than migraine (epilepsy, arterial hypertension, heart failure and/or depression).
2.  < 2 prescriptions during the follow-up period.
3. No active representation in the database for 12 months before and/or after the index date.
4.  < 2 registered data entries in the database.
5. Transfer to another centre, displacement or out of zone.
6. Permanent institutionalization.
7. Terminal illness, human immunodeficiency virus (HIV) or need for dialysis.

The study was approved by the Ethics Committee of the Consorci Sanitari de Terrassa (Terrassa, Spain) on 27/01/2020. Written informed consent was not requested as information obtained from patient medical records was dissociated from the personal identification data in accordance with the current General Data Protection Regulation 2016/679 of the European Parliament (EU-GPDR) and the Council of 27 April 2016 and with the Spanish Organic Law 3/2018 of 5 December 2018 on Data Protection and Guarantee of Digital Rights.

Due to the retrospective nature of the study, there was no influence on any decision regarding medication prescription.

### Description of the variables and outcome measures

#### Demographic and clinical variables

Demographic and clinical information at index date, extracted from the patient medical records, included: age, gender, body mass index (BMI), time since diagnosis (diagnosis date – index date), comorbidities and smoking status. To describe the implication of co-occurring conditions in every patient, we calculated the mean number of chronic comorbidities as well as the Charlson Comorbidity Index (CCI) [24] as a surrogate of severity.

#### Preventive, symptomatic and concomitant pharmacological treatments

Information on migraine preventive and symptomatic drugs as well as other concomitant treatments was collected during the follow-up period and classified according to the Anatomical Therapeutic Chemical (ATC) system [25]. Prescription data, including dates and type of prescriber (i.e., primary care, neurologist, etc.) were obtained from pharmacy claims files.

Preventive treatments were collected from index date to end of follow-up (12 months). Data on symptomatic and concomitant prescriptions were additionally obtained during the pre-index period. Botulinum toxin prescriptions were not available in the The Big-Pac™ database.

#### Healthcare resource use, sick leave and associated costs

HRU collected during the one-year follow-up period included health services (primary care and specialist visits, A&E admissions and hospitalization), medical tests (laboratory and other tests, conventional radiology, computed tomography scan and magnetic resonance imaging) and pharmacological treatments. Additionally, days of sick leave were extracted to describe patients’ loss of labour productivity.

These variables were used to calculate associated direct healthcare costs (healthcare services, medical tests and pharmacological prescriptions) and non-healthcare costs (indirect; related to loss of work productivity) during the study period. All economic information regarding medical care was obtained from the centres’ analytical accounting (2019). The medical prescriptions were quantified according to the public retail price per pack at the time of prescription (according to Bot Plus from the General Council of the Spanish Official College of Pharmacists) [26]. Indirect costs were calculated with the mean interprofessional wage information at the time [27]. Other non-healthcare out-of-pocket expenses were not recorded in the database and were hence excluded from the present analysis. Costs were expressed as an average cost per patient (average / unit) per year (€, 2019).

#### Definitions and outcomes measures

To analyze the persistence to available treatments for migraine prevention in Spain and to describe the prescription patterns the following outcomes were calculated:

- Persistence time or duration of treatment, including:
  - ◦ The elapsed time from the index date until treatment discontinuation or the end of the study, whichever came first. Discontinuation implied either permanent treatment dropout or reinitiation of the same or another treatment (after 60 days). Treatment switching between different prophylactic medications or dose increase/decrease within 60 days or less since the last prescription were not considered discontinuation events since it is common clinical practice [28]. The 60-day gap period was defined in accordance with other publications and expert opinion.
  - ◦ 6- and 12-month persistent rates: percentage of patients who persisted with their preventive treatment until day 183 and day 365 after the index date. Persistence was defined as the prescribed treatment continuation or switching during the study follow-up period, either with or without dose adjustments.
- Prescription patterns:
  - ◦ Switching events: percentage of patients who changed their preventive medication within 60 days from the last prescription. Reinitiation events: percentage of patients who restarted the same or another medication after a gap of 60 days or more since the last prescription.

To assess the relationship between persistence to preventive treatment and HRU and related costs, patients were divided into two study groups according to their persistence at 12 months (persistent vs. non-persistent patients). The difference in annual HRU and costs between the two groups was then calculated. Additionally, we explored the factors that could be associated to treatment persistence or non-persistence by comparing them in both groups. These included the demographic variables described above, the patient comorbidities and the type of initial preventive medication prescriber.

### Statistical analysis

Demographics and clinical characteristics were summarized for the two groups (persistent and non-persistent) using descriptive statistics. Number of patients and percentages are provided for categorical variables and mean, standard deviation and 95% confidence intervals for the mean for continuous variables. To better represent the skewness of cost and HRU variables, median and interquartile range (IQR) were also presented. For HCRU and costs variables, differences between groups are summarized with mean and SD and associated 95% 2-sided CI for the mean differences. Additionally, a sensitivity analysis with an ANCOVA model (analysis of covariance) has been performed to adjust the difference in costs for the following confounders: gender, age, CCI and time since diagnosis. IBM’s Statistical Package for the Social Sciences (SPSS®) for Windows version 23 was used.

**Fig. 1:**
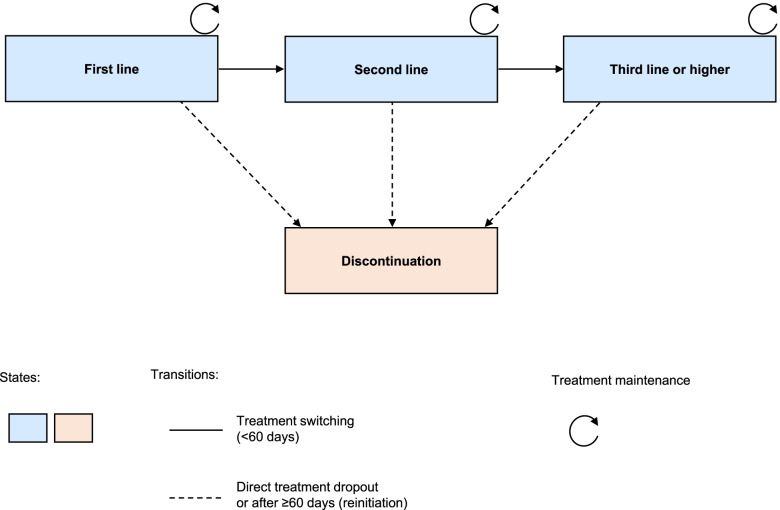
Multi-state model design of the migraine preventive therapy. To create the model, it was assumed that patients in any line of treatment could maintain it, discontinue it or switch medications, which generated four possible states and five different transitions. Treatment switching was defined by any change of medication within 60 days from the last prescription, while treatment discontinuation may have been due to reinitiation after 60 days or more without renovating it or direct mediation dropout

A Multi-state Cox model (MSM) of proportional hazards [29] was applied to compare the discontinuation rates among the different treatment lines as well as the influence of the covariates age, gender, CCI and time since diagnosis. Discontinuation in the first-line treatment was estimated via Kaplan-Maier and reported with median and 95% confidence interval (CI). Results were reported as hazard ratios (HR) or 5-year HR (when the values were smaller than 1). The MSM allows the inclusion of intermediate states and to compare the discontinuation rate between initial and following states. Four possible states were assumed, which corresponded to the first, second and third (or higher) lines of treatment as well as discontinuation. At each state, patients could continue, discontinue or switch treatments (Fig. 1). The MSM was designed with the mstate and survival packages in R [30].

The analyses were done with the data from all patients that met the eligibility criteria.

## Results

### Study population and baseline characteristics

**Fig. 2:**
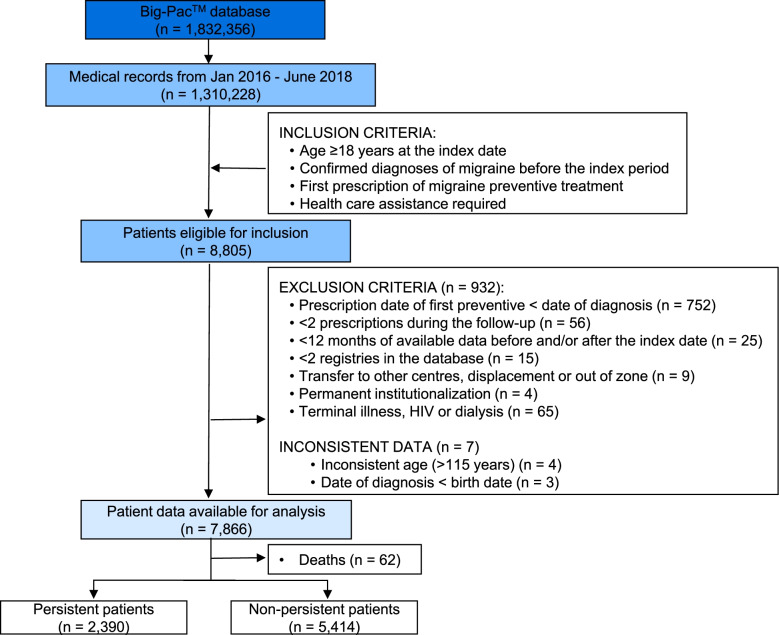
Study flow chart. Filtering data entries in the Big-Pac™ database by study period yielded 1,310,228 patients. Eligible patients under preventive treatment of migraine were identified and split into two study groups

The Big-Pac™ database contained information of 1,832,356 patients. Out of 8,805 eligible patients, 939 were excluded and 7,866 were analyzed (Fig. 2). A total of 62 (0.8%) deaths occurred during the study follow-up; these patients were not included in the subgroups of persistence and non-persistence patients (Fig. 2).

Table 1 shows the baseline characteristics of the study population. The mean age was 48.2 (SD: 14.8) years, and we observed a large majority of women (6,324; 80.4%). The mean BMI was 27.8 (SD: 4.5) and the CCI value was 0.4 (SD: 0.9). We found that the most frequent comorbidities were generalized anxiety disorder (2,827 patients; 35.9%), dyslipidaemia (2,124; 27.0%), arterial hypertension (1,724; 21.9%) and smoking (1,426; 18.1%).

**Table 1:** Demographic and clinical characteristics of migraine patients at baseline and in non-persistent and persistent study groups

	Entire study population *N* = 7,866 (100%)	Non-persistent patients *N* = 5,414 (69.4%)	Persistent patients *N* = 2,390 (30.6%)
**Age**, years			
Mean (SD)	48.2 (14.8)	47.6 (14.7)	48.8 (14.3)
**Age range**, n (%)			
18—44 years	3,296 (41.9%)	2,343 (43.3%)	949 (39.6%)
45—64 years	3,527 (44.8%)	2,384 (44.1%)	1,127 (47.1%)
65—74 years	713 (9.1%)	479 (8.9%)	223 (9.3%)
≥ 75 years	330 (4.2%)	204 (3.8%)	95 (4.0%)
**Gender (women)**, n (%)	6,324 (80.4%)	4,361 (80.6%)	1,918 (80.3%)
**BMI**, kg/m^2^			
Mean (SD)	27.8 (4.5)	27.7 (4.5)	27.9 (4.3)
**Time since diagnosis**, years^a^			
Mean (SD)	3.6 (1.9)	3.6 (1.9)	3.7 (1.9)
**Comorbidities per patient**			
Mean (SD)	1.6 (1.4)	1.6 (1.4)	1.6 (1.4)
**CCI**			
Mean (SD)	0.4 (0.9)	0.4 (0.9)	0.4 (0.9)
**Comorbidities**, n (%)			
Generalized anxiety disorder	2,827 (35.9%)	1,968 (36.4%)	813 (34.0%)
Dyslipidaemia	2,124 (27.0%)	1,445 (26.7%)	648 (27.1%)
Arterial hypertension	1,724 (21.9%)	1,170 (21.6%)	524 (21.9%)
Obesity	1,161 (14.8%)	794 (14.7%)	353 (14.8%)
Depressive syndrome	890 (11.3%)	607 (11.2%)	272 (11.4%)
Asthma	622 (7.9%)	450 (8.3%)	166 (6.9%)
Malignant neoplasms	620 (7.9%)	439 (8.1%)	168 (7.0%)
Diabetes	529 (6.7%)	397 (7.3%)	118 (4.9%)
COPD	220 (2.8%)	154 (2.8%)	63 (2.6%)
Stroke	170 (2.2%)	112 (2.1%)	55 (2.3%)
Ischaemic heart disease	115 (1.5%)	69 (1.3%)	41 (1.7%)
Kidney failure	121 (1.5%)	85 (1.6%)	30 (1.3%)
Heart failure	99 (1.3%)	62 (1.1%)	31 (1.3%)
**Other risk factors**, n (%)			
Active smoking	1,426 (18.1%)	922 (17.0%)	478 (20.0%)

*BMI* body mass index, *CCI* Charlson Comorbidity Index, *COPD* chronic obstructive pulmonary disease, *SD* standard deviation

^a^The Big-Pac™ database contains data from 2012 onwards. For patients diagnosed before 2012, we assumed the first entry in the database as the date of diagnosis

The group of persistent patients resulted in a smaller number of patients (2,390; 30.6%) than the non-persistent one (5,414; 69.4%).

Difference in demographic variables and frequency of comorbidities between persistent and non-persistent were minor (Table 1). In non-persistent patients the incidence of diabetes (397; 7.3% vs. 118; 4.9%), asthma (450; 8.3% vs. 166; 6.9%) and generalized anxiety disorder (1,968; 36.4% vs. 813; 34.0%) was slightly higher and the frequency of active smokers was lower (922; 17.0% vs. 478; 20.0%) compared with the persistent ones.

### Prescription of migraine treatments and involved healthcare professionals

First-time preventive treatment prescribers were mostly primary care physicians (4,093; 75.6% vs. 1,896; 79.3%) followed by neurologists (893; 16.5% vs. 324; 13.5%) for both non-persistent and persistent patients, respectively (Table 2).

**Table 2:** Prescription of first migraine preventives by healthcare professionals

	Non-persistent patients *N* = 5,414 (69.4%)	Persistent patients *N* = 2,390 (30.6%)
**Medical specialty or service**, n (%)		
Primary Care	4,093 (75.6%)	1,896 (79.3%)
Neurology	893 (16.5%)	324 (13.5%)
Psychiatry	146 (2.7%)	62 (2.6%)
A&E	65 (1.2%)	24 (1.0%)
Internal Medicine	27 (0.5%)	12 (0.5%)
Others	190 (3.5%)	72 (3.0%)

*A&E* accident & emergency department

At baseline, antidepressants (3,642; 46.3%), antiepileptics (1,738; 22.1%) and beta-blockers (1,399; 17.8%) were the most frequently prescribed drugs for migraine prevention (Supplementary Fig. 2).

**Fig. 3:**
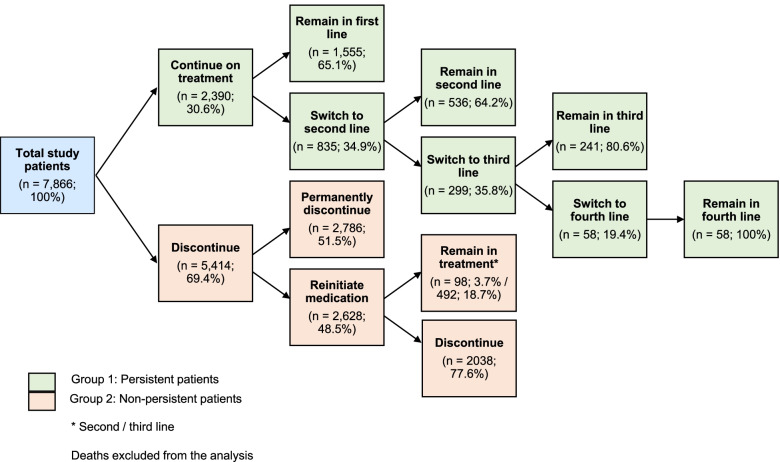
Flow diagram of treatment evolution in the two study groups. Persistent patients continued on their migraine preventive medication during the 12-month follow-up period or switched to another line of treatment within 60 days from the last prescription. Non-persistent patients permanently discontinued their preventive treatment or restarted it after 60 days

Up to four lines of preventive treatments (switches) were observed in the persistent group during the 12-month study period. Non-persistent patients showed a maximum of three lines of treatment (reinitiations) during the same period (Fig. 3; Supplementary Fig. 1).

The acute treatments prescribed on the index date were NSAIDs (3,142; 58.0% of non-persistent vs. 1,388; 58.1% of persistent patients), triptans (1,525; 28.2% vs. 692; 29.0%, respectively) and ergotics (79; 1.5% vs. 37; 1.6%).

### Healthcare resource use and associated costs

Non-persistent patients required more primary care (mean (SD): 12.0 (8.8) vs. 9.0 (8.7)), specialist (mean (SD): 2.2 (1.1) vs. 1.1 (0.9)) and emergency room (mean (SD): 1.3 (2.2) vs. 0.5 (1.2)) visits than those who persisted with their treatment, respectively. Thus, the mean number of primary care visits, specialist visits and A&E admissions per year was respectively 3.0, 1.1 and 0.9 times higher in the non-persistent group compared with persistent patients. The number of days of sick leave per patient and year was also higher in non-persistent subjects (mean difference: 2.7; 95% CI: 0.8;4.5) (Table 3).

**Table 3:** Use of healthcare resources

	Non-persistent patients *N* = 5414 (69.4%)	Persistent patients *N* = 2390 (30.6%)	Difference (Persistent – Non-persistent) *N* = 7866
**Primary Care visits**			
- Mean (SD)	12.0 (8.8)	9.0 (8.7)	-2.98
- CI 95%	(11.8;12.2)	(8.7;9.4)	(-3.41; -2.56)
- Median (P25–P75)	10 (6–15)	6 (3–12)	
- Min-Max	0–59	0–59	
**Specialist visits**			
- Mean (SD)	2.2 (1.1)	1.1 (0.9)	-1.07
- CI 95%	(2.1;2.2)	(1.1;1.1)	(-1.12; -1.01)
- Median (P25–P75)	2 (1–3)	1 (1–1)	
- Min-Max	0–10	0–10	
**A&E admissions**			
- Mean (SD)	1.3 (2.2)	0.5 (1.2)	-0,86
- CI 95%	(1.3;1.4)	(0.4;0.5)	(-0.95; -0.76)
- Median (P25–P75)	0 (0–5)	0 (0–0)	
- Min-Max	0–5	0–5	
**Hospital stays**			
- Mean (SD)	0.2 (1.4)	0.1 (1.3)	-0.03
- CI 95%	(0.1;0.2)	(0.1;0.2)	(-0.1; 0.03)
- Median (P25–P75)	0 (0–0)	0 (0–0)	
- Min-Max	0–44	0–22	
**Laboratory tests**			
- Mean (SD)	1.4 (1.2)	0.7 (1.1)	-0.67
- CI 95%	(1.4;1.4)	(0.7;0.8)	(-0.72; -0.61)
- Median (P25–P75)	1 (0–2)	0 (0–1)	
- Min-Max	0–4	0–4	
**Conventional radiology**			
- Mean (SD)	1.1 (1.5)	1.0 (1.4)	-0.08
- CI 95%	(1.0;1,1)	(0.9;1.0)	(-0.15: -0.01)
- Median (P25–P75)	0 (0–2)	0 (0–2)	
- Min-Max	0–5	0–5	
**CT scan**			
- Mean (SD)	0.1 (0.3)	0.1 (0.2)	-0.02
- CI 95%	(0.1;0.1)	(0;0.1)	(-0.03;0)
- Median (P25–P75)	0 (0–0)	0 (0–0)	
- Min-Max	0–3	0–2	
**MRI**			
- Mean (SD)	0.1 (0.4)	0.1 (0.4)	-0.01
- CI 95%	(0.1;0.1)	(0.1;0.1)	(-0.03;0.01)
- Median (P25–P75)	0 (0–0)	0 (0–0)	
- Min-Max	0–5	0–5	
**Other tests**			
- Mean (SD)	1.2 (1.2)	1.0 (1.1)	-0.16
- CI 95%	(1.2;1.2)	(1.0;1.1)	(-0.22; -0.1)
- Median (P25–P75)	1 (0–2)	1 (0–2)	
- Min-Max	0–5	0–5	
**Days of sick leave**			
- Mean (SD)	14.7 (38.8)	12.0 (37.4)	-2.69
- CI 95%	(13.7;15.8)	(10.5;13.5)	(-4.54; -0.84)
- Median (P25–P75)	1 (1–11)	0 (0–4)	
- Min-Max	0–365	0–365	

*A&E* accident & emergency, *CI* confidence interval, *CT* computed tomography, *MRI* magnetic resonance imaging, *SD* standard deviation

In terms of costs, the average expenditure was €2,345 (SD: 4,189) per patient per year in the persistent group, which represented a saving of €-622 (95% CI: -829; -415) compared to those who discontinued their preventive treatment (€2,967; SD: 4,353) (Table 4). This difference was due to an increase in both the use of healthcare resources and medication (mean difference: €-350; 95% CI: -421; -278) as well as non-healthcare indirect costs (mean difference: €-272; 95% CI: -459; -85) (Table 4).

**Table 4:** Annual healthcare and non-healthcare costs associated with migraine patient management and preventive treatment persistence

	Non-persistent patients *N* = 5414 (69.4%)	Persistent patients *N* = 2390 (30.6%)	Difference (Persistent – Non-persistent) *N* = 7866
***Health services***^a,^ € patient-year			
**Primary Care visits**,			
Mean (SD)	278.1 (204.1)	208.9 (200.9)	-69.2
95% CI			(-79.0; -59.4)
**Specialist visits**			
Mean (SD)	199.4 (104.4)	100.7 (87.8)	-98.7
95% CI			(-103.5; -93.9)
**A&E admissions**			
Mean (SD)	156.9 (259.2)	56.2 (135.3)	-100.7
95% CI			(-111.7; -89.7)
**Hospital stays**			
Mean (SD)	76.2 (681.4)	59.8 (628.0)	-16.5
95% CI			(-48.5; 15.6)
***Medical tests***^b,^ € patient-year			
**Laboratory tests**			
Mean (SD)	45.2 (38.7)	23.6 (34.4)	-21.6
95% CI			(-23.4; -19.8)
**Conventional radiology**			
Mean (SD)	30.2 (41.4)	28.0 (40.2)	-2.2
95% CI			(-4.2; -0.2)
**CT scan**			
Mean (SD)	6.4 (26.0)	4.9 (22.5)	-1.5
95% CI			(-2.7; -0.3)
**MRI**			
Mean (SD)	21.7 (77.0)	19.5 (72.4)	-2.2
95% CI			(-5.9; 1.4)
**Other tests**			
Mean (SD)	55.9 (57.0)	48.4 (50.4)	-7.5
95% CI			(-10.2; -4.9)
***Medication***^c,^ € patient-year			
**Symptomatic medication**			
Mean (SD)	254.8 (769.4)	257.7 (933.3)	2.9
95% CI			(-36.7; 42.5)
**Preventive medication**			
Mean (SD)	155.9 (368.1)	152.3 (373.6)	-3.5
95% CI			(-21.3; 14.3)
**Concomitant medication**			
Mean (SD)	162.0 (389.4)	141.5 (437.9)	-20.5
95% CI			(-40.0; -1.0)
**Total healthcare direct costs**, € patient-year			
Mean (SD)	1,476.8 (1,472.9)	1,127.1 (1,521.4)	-349.8
95% CI			(-421.4; -278.1)
**Non-healthcare indirect costs**, € patient-year^d^			
Mean (SD)	1,490.0 (3,930.0)	1,217.7 (3,786.4)	-272.3
95% CI			(-459.4; -85.2)
**Total costs**, € patient-year			
Mean (SD)	2,966.8 (4,352.6)	2,344.8 (4,189.1)	-622.1
95% CI			(-829.2; -414.9)
**Adjusted costs**, € patient-year^e^			
Mean	2,929.0	2,346.0	-583.0
(95% CI)	(2,784.0;3,073.0)	(2,130.0;2,562.0)	(-708.0; -458.0)

*CCI* Charlson Comorbidity Index, *CI* confidence interval, *CT* computed tomography, *MRI* magnetic resonance imaging, *SD* standard deviation, *VAT* Value-Added Tax

^a^Unit costs as per the 2019 analytical accounts: Primary Care visit: €23.2; specialist visit (Neurology, Internal Medicine and Anaesthesiology only): €92.5; A&E admission: €117.5; overnight hospital stay: €480.9; pain clinic visit: €185.0

^b^Unit costs as per the 2019 analytical accounts: laboratory tests: €32.3; conventional radiology: €28.5; CT scan: €96.0; MRI: €177.0; other diagnostic tests: €47.1

^c^Unit costs including VAT at the time of prescription

^d^Unworked day cost: €101.21

^e^ANCOVA model, least square means corrected for the following covariates: gender, age, CCI and time since diagnosis

The difference in costs after adjusting for the covariates: gender, age, CCI and time since diagnosis resulted in similar savings per patient per year: €-583, 95% CI: (-708; -458).

### Persistence and risk of discontinuation to migraine preventive treatment

The first-line median persistence time was 162 days (95% CI: 158–166). The reduced number of treatment dropouts from the other lines together with the different treatment period in each line do not allow the estimation of their corresponding median persistence. Patients were treated with preventives for a mean period of 166.7 (SD: 136.9) days and a median of 86 (IQR: 57–365) days, including treatment line switching. The persistence rates at 6 (2,545 patients; 32.4%) and 12 (2,390; 30.4%) months were similar.

Among persistent patients (2,390; 30.6%), 1,555 (65.1%) maintained their first-line therapy or switched to a second (835; 34.9%), third (299; 35.8%) or fourth (58; 19.4%) line of treatment. After treatment switching, 536 (64.2%), 241 (80.6%) and 58 (100%) patients maintained their second, third or fourth line of treatment until the end of the observation period, respectively. Among non-persistent patients (5,414; 69.4%), 2,786 (51.5%) permanently discontinued their medication. The other 2,628 (48.5%) patients restarted the same or another one after 60 days or more since the last prescription. After reinitiation, 98 (3.7%) and 492 (18.7%) patients remained in the second or third line, respectively, and 2,038 (77.5%) patients finally discontinued their medication (Fig. 3). Further details on treatment switching and reinitiation events are shown in Supplementary Fig. 1.

In the assessment of which therapeutic line could be possibly associated with a higher risk of discontinuation, we observed a lower risk of discontinuation in the second or third line (or higher) of preventive treatment than in the first one, with a statistically significant reduction in the HR of the second line compared to the first one (HR: 0.24; 95% CI: 0.1;0.5; *P* < 0.001 and HR: 0.21; 95% CI: 0.0;1.4; *P* = 0.11, respectively) (Table 5 and Supplementary Fig. 3). Additionally, age was significantly associated with an increased risk of abandoning the treatment (5-year HR: 5.06 and 5.1; *P* < 0.001 for the first and second lines, respectively) (Table 5).

**Table 5:** Multi-state model for the migraine preventive treatment: risk of discontinuation and analyzed covariates

	**From the 1st line**			**From the 2nd line**			**From the 3rd or higher**		
	HR	SD	*P*	HR	SD	*P*	HR	SD	*P*
**Age**^**a**^	**5.06**	** < 0.001**	** < 0.001**	**5.10**	** < 0.001**	** < 0.001**	5.09	0.01	0.237
**Gender**	0.98	0.03	0.526	0.87	0.19	0.455	1.09	0.55	0.882
**CCI**	1.01	0.01	0.516	0.95	0.09	0.582	1.01	0.28	0.96
**Time since diagnosis**	0.99	0.01	0.048	0.97	0.04	0.432	1.04	0.11	0.730
**Discontinuation (vs. 1st line)**	n/a			**0.24**	**0.35**	** < 0.001**	0.21	0.98	0.110

*CCI* Charlson Comorbidity Index, *HR* hazard ratio, *n/a* not applicable, *SD* standard deviation, *P* *p*-value

^a^5-year HR

## Discussion

This is the first study presenting the costs savings associated to the sustained preventive treatment of migraine in a large sample managed in a real world setting of the European Union. Persistence to traditional oral migraine prophylaxis was poor, especially in the first half-year, and migraine-associated costs were higher for non-persistent patients. In this study, we compared the cohort of persistent and non-persistent patients and analyzed the risk of preventive treatment discontinuation according to several factors.

When indicated, preventive treatment and persistence to it can help achieve the therapeutic goals and have economic benefits for public healthcare systems [10, 17, 21, 22]. Our results point towards the same direction. Non-persistent patients require more medical assistance and days of sick leave compared with persistent ones. The costs also increased when patients dropped out of treatment, with a mean total cost difference of €622 per patient per year. In Europe and the USA, indirect costs represent most of the economic burden associated with migraine, which is mainly derived from impairments in productive work [9]. In our sample, indirect costs in each study group represented approximately half of the total expenditure, which coincides with the Spanish 2018 atlas of migraine [31, 32]. Regarding healthcare direct costs, our estimated expenses (€1,127 to €1,477 depending on treatment persistence/non-persistence, respectively) also fall in between reference ranges for episodic (€964—€1,092 per patient/year) and chronic migraine (2,670 – 3,847 per patient/year) in Spain [31, 32]. In most European countries, active workers, pensioners or people perceiving unemployment benefits as well as their relatives are entitled to full health cover, an allowance for temporary incapacity and part or complete reimbursement of public medical prescriptions [33]. Thus, the aforementioned cost difference between patient groups has a direct impact on the public purse, which represents an important factor to be considered when comparing health economic studies across countries, especially those without a similar public system [34, 35]. These data support the claim that an association between preventive treatment discontinuation and subsequently increased medical care and economic burdens. Hence, the application of healthcare policies reinforcing the use of prophylactic therapies that may contribute to alleviate them.

Our results on persistence to preventive treatment are more optimistic than those presented by Hepp and colleagues, who found 6- and 12-month persistence rates to oral preventive medication of 25% and 14%, respectively [28]. Nonetheless, we suspect that these differences may be at least partially explained by the inclusion of patients with chronic migraine only and the definition of persistence used (≤ 30 days between consecutive prescriptions), which excluded the patients that switched [28]. In our study, patients that switched their medication within 60 days after the previous prescription were also considered persistent apart from the ones who maintained the same line of treatment. Another pooled analysis of observational studies found that persistence to propranolol, amitriptyline, and topiramate can range from 19 to 79% at 6 months, and 7 to 55% at 12 months, which could also be explained by methodological discrepancies [19]. In sum, although results from different studies need to be interpreted cautiously, our conclusions are in line with the available evidence on migraine preventive treatment. Overall, it shows poor adherence and persistence to migraine prophylaxis.

Regarding the type of medication, prescription preferences in different countries may be subject to multiple variables such as local practice, availability, costs and reimbursement policies. For example, flunarizine was used as the first preventive treatment in almost a tenth of patients in our study, whereas in the US and Japan it is not available by prescription. Also, we found that the antidepressant amitriptyline was the most common prescription for migraine prophylaxis, though it was ranked level B of evidence for efficacy (i.e. probably effective) by the American Academy of Neurology and the American Headache Society, and it has been recently recommended as a second-line medication by several European medical societies [15, 36]. In contrast, topiramate and propranolol, which are the front-line recommended therapies for migraine prevention among others [15, 36], ranked second and third in our list. Finally, the fact that only 58% and 28% of patients in each study group received symptomatic treatment with NSAIDs and triptans is intriguing and may be a sign of poor disease management since the expected percentages fell well above 90% and 80%, respectively. Nonetheless, our results coincide with a national survey that assessed the treatment preferences for migraine among Spanish neurologists carried out in 2018 [31, 32]. In the case of botulinum toxin, the number of patients that may have received it upon oral preventive treatment failure was unknown since it is not currently dispensed by community pharmacies in our country. Other types of treatments that we may have disregarded are the over-the-counter ones and those prescribed by paper and/or private medical care.

When characterizing the profile of our study population, we observed that almost half of our patients commonly experienced psychiatric comorbidities such as generalized anxiety disorder and depressive syndrome. According to previous works, they can interfere with migraine evolution and treatment outcomes [33–39], so special consideration should be taken towards them, not only as reactive symptoms but also as migraine chronification.

For episodic migraine, Spanish official guidelines recommend maintaining migraine preventive treatment for 6 months, with a minimal period of 3 months and a maximum that depends on individual clinical features [40]. Other European and American recommendations suggest a minimum trial period of 2–3 months to achieve efficacy [2, 15]. Therefore, the first half-year of our study was key. After six months, only a third of migraine patients were still on preventive treatment and the highest dropout rate was observed during the first 3 months.

The MSM results showed that medication discontinuation could be state-dependent, as suggested by the different HR values obtained from each treatment line. In this sense, no other previous works in migraine patient management have addressed differential discontinuation risks across therapeutic lines and how patients transition among them. In this work, we have observed that the probability of abandoning the treatment was higher during the first line compared to the second one. The same trend was observed for the treatments administered in third line or higher, although it did not reach statistical significance, probably due to the lower number of patients in this category. This different discontinuation risk may have been related to different plausible therapeutic behaviours: 1) Patients followed tapered withdrawal for some time according to guideline recommendations [2, 11, 15] or, on the contrary, 2) patients permanently discontinued their medication due to poor efficacy, safety, tolerability or satisfaction with the treatment. In previous studies, lack of efficacy and tolerability/safety issues were the most frequent reasons for discontinuation according to previous works [17, 19, 21, 41]. However, other reasons, such as patients lost to follow-up or patient’s choice (including reasons related to cost/insurance, access, travel, etc.), have been reported too [17, 19, 41], which should be revisited to increase persistence rates. Our results suggest that patients taking their first oral medication may be especially susceptible to long-term discontinuation, and increasing the awareness on the possibility of a second, third or even a fourth line of therapy may increase the persistence rate. Finally, more data regarding clinical characteristics may be needed to investigate whether patients that cycle through different lines of treatments suffer from a more severe migraine and a higher need to improve their symptoms.

Despite the high number of patients analyzed, the retrospective nature of this work imposes some limitations such as the disease underreporting, possible misdiagnosis codes, other non-reported comorbidities by the patients or any other missing data. Regarding a possible bias in the estimation of the discontinuation risk, subjects in second or higher line of therapy, do not have the chance to stay on treatment as long as first line, resulting in a decrease of the HR. However, we have observed that most of the discontinuations happened within the first 6 months leaving as much time for at least a second line therapy as it was for the first line.

Future studies should address these limitations and include CGRP antagonists for migraine prophylaxis, which were marketed in Spain after our study period [42]. Despite their high cost and restricted availability, their ease of use (monthly or quarterly administration) is expected to increase treatment persistence [2, 14, 43].

## Conclusion

In our study, only around 30% of people living with migraine who were prescribed prophylaxis for the first time maintained their treatment after 6 months. Patients with persistent treatment for one year used fewer healthcare resources and implied lower annual expenditure, suggesting that the benefit of preventive treatments goes beyond the impact on pain.

## Supplementary Information

**Additional file 1: Figure S1.** Flow diagram of migraine preventive treatment persistence and discontinuation throughout one year of follow-up. The number of persistent or non-persistent patients as well as the percentage in respect to the total number of patients are indicated in brackets. Persistent patients maintained their medication until the end of the study or change it within 60 days from the last prescription (switching). Non-persistent patients dropped it out permanently or restarted another line of treatment 60 days or more after the last prescription (reinitiation).

**Additional file 2: Figure S2.** Front-line migraine preventive treatments. *N* = 7,866 patients.

**Additional file 3: Figure S3.** Multi-state model treatment curves. The graph shows the distribution of the proportions of patients in the different states at any time of the study. Persistence curves for the second and third (or higher) lines of treatment remained similar and stable throughout the study. In contrast, first-line persistence curve decreased and was steeper in the first 6 months (and even more in the first three) as a sign of a higher discontinuation rate in this time window.

## Data Availability

The data that support the findings of this study are available from ATRYS HEALTH, SA but restrictions apply to the availability of these data, which were used under license for the current study, and so are not publicly available. Data are however available from the authors upon reasonable request and with permission of ATRYS HEALTH, SA.

## Abbreviations

ACE: Angiotensin-converting enzyme
ARBs: Angiotensin II receptor blockers
ATC: Anatomical Therapeutic Chemical
A&E: Accident & emergency department
BMI: Body mass index
CCI: Charlson Comorbidity Index
CGRP: Calcitonin gene-related peptide
CI: Confidence interval
CT: Computed tomography
EU-GPDR: General Data Protection Regulation 2016/679 of the European Parliament
HIV: Human immunodeficiency virus
HR: Hazard ratio
HRU: Healthcare resource use
ICD-10-CM: International Classification of Diseases (10^th^ edition) Clinical Modification
IQR: Interquartile range
MRI: Magnetic resonance imaging
MSM: Multi-state model
NSAIDs: Nonsteroidal anti-inflammatory drugs
SD: Standard deviation
VAT: Value-Added Tax
