# Supplementary figures and images for: Persistence, use of resources and costs in patients under migraine preventive treatment: the PERSEC study
Source: J Headache Pain. 2022 Jul 7;23(1):78. doi: 10.1186/s10194-022-01448-2 (PMC9261063; doi:10.1186/s10194-022-01448-2)

## Slide 1
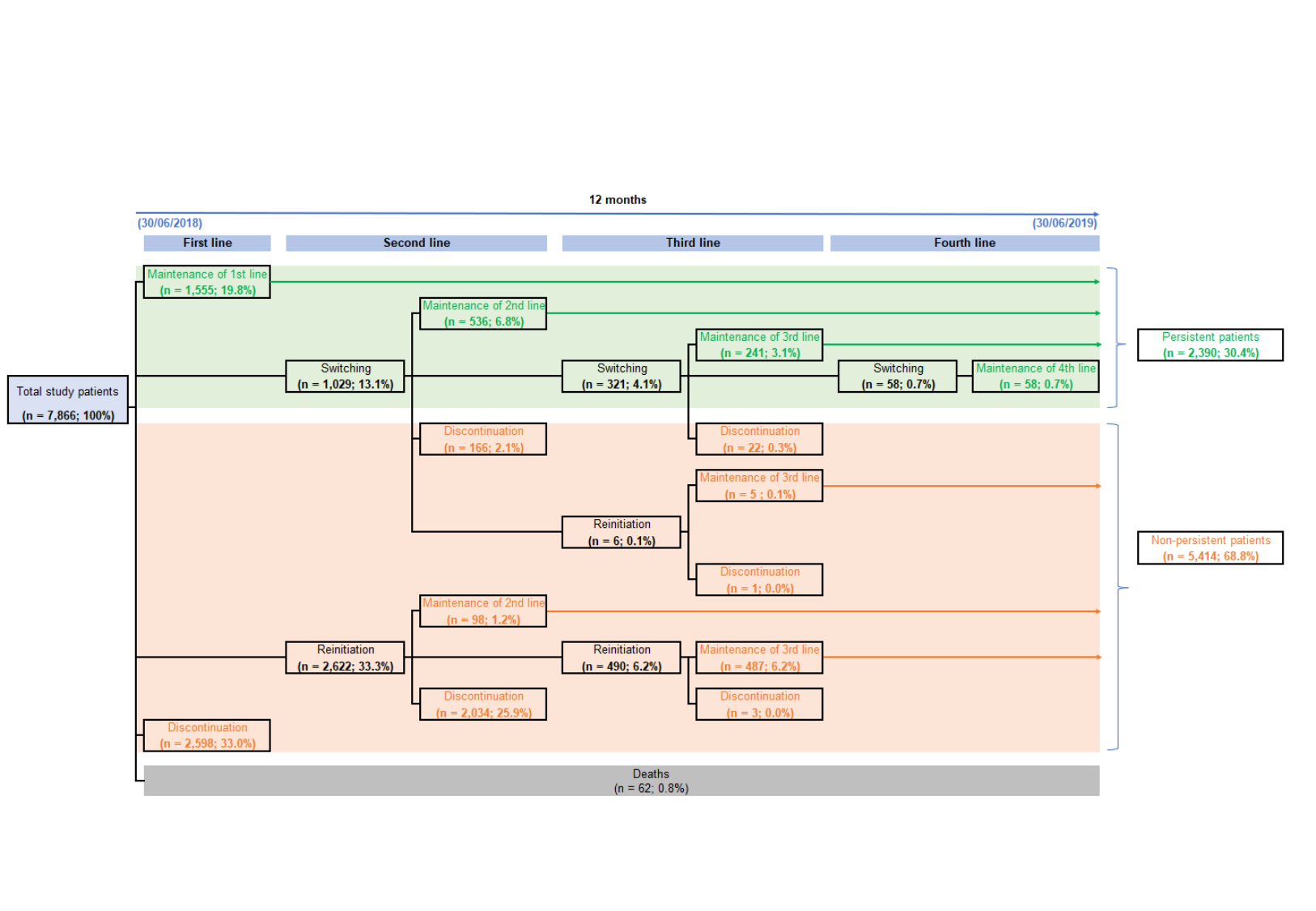

Supplement: Supplementary file 1 — Additional file 1: Figure S1. Flow diagram of migraine preventive treatment persistence and discontinuation throughout one year of follow-up. The number of persistent or non-persistent patients as well as the percentage in respect to the total number of patients are indicated in brackets. Persistent patients maintained their medication until the end of the study or change it within 60 days from the last prescription (switching). Non-persistent patients dropped it out permanently or restarted another line of treatment 60 days or more after the last prescription (reinitiation). [file 10194_2022_1448_MOESM1_ESM.pptx]

## Slide 1
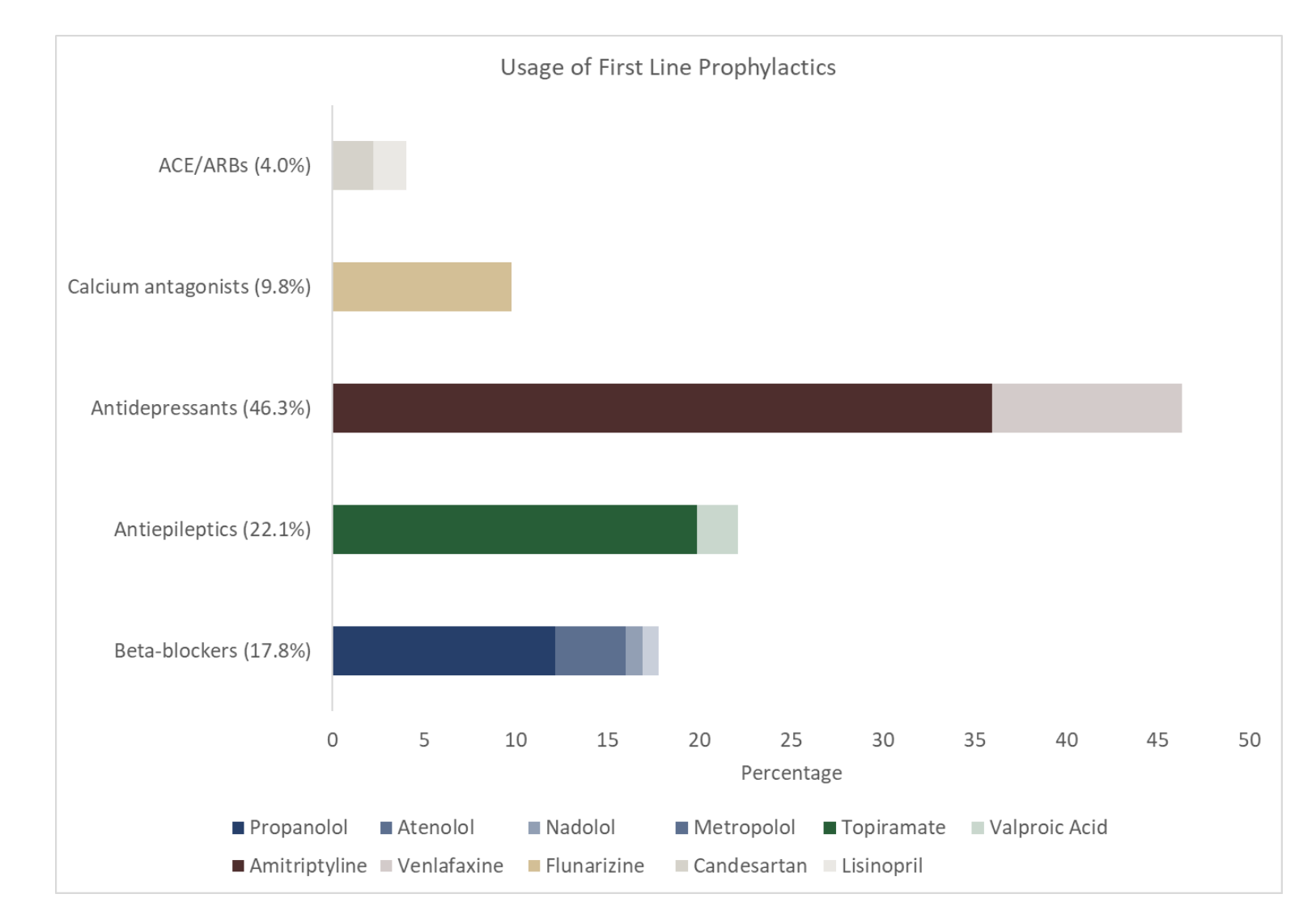

Supplement: Supplementary file 2 — Additional file 2: Figure S2. Front-line migraine preventive treatments. N = 7,866 patients. [file 10194_2022_1448_MOESM2_ESM.pptx]
