# Supplementary material for: Persistence, use of resources and costs in patients under migraine preventive treatment: the PERSEC study
Source: J Headache Pain. 2022 Jul 7;23(1):78. doi: 10.1186/s10194-022-01448-2 (PMC9261063; doi:10.1186/s10194-022-01448-2)
Supplement: Supplementary file 3 — Additional file 3: Figure S3. Multi-state model treatment curves. The graph shows the distribution of the proportions of patients in the different states at any time of the study. Persistence curves for the second and third (or higher) lines of treatment remained similar and stable throughout the study. In contrast, first-line persistence curve decreased and was steeper in the first 6 months (and even more in the first three) as a sign of a higher discontinuation rate in this time window. [file 10194_2022_1448_MOESM3_ESM.pptx]

## Slide 1
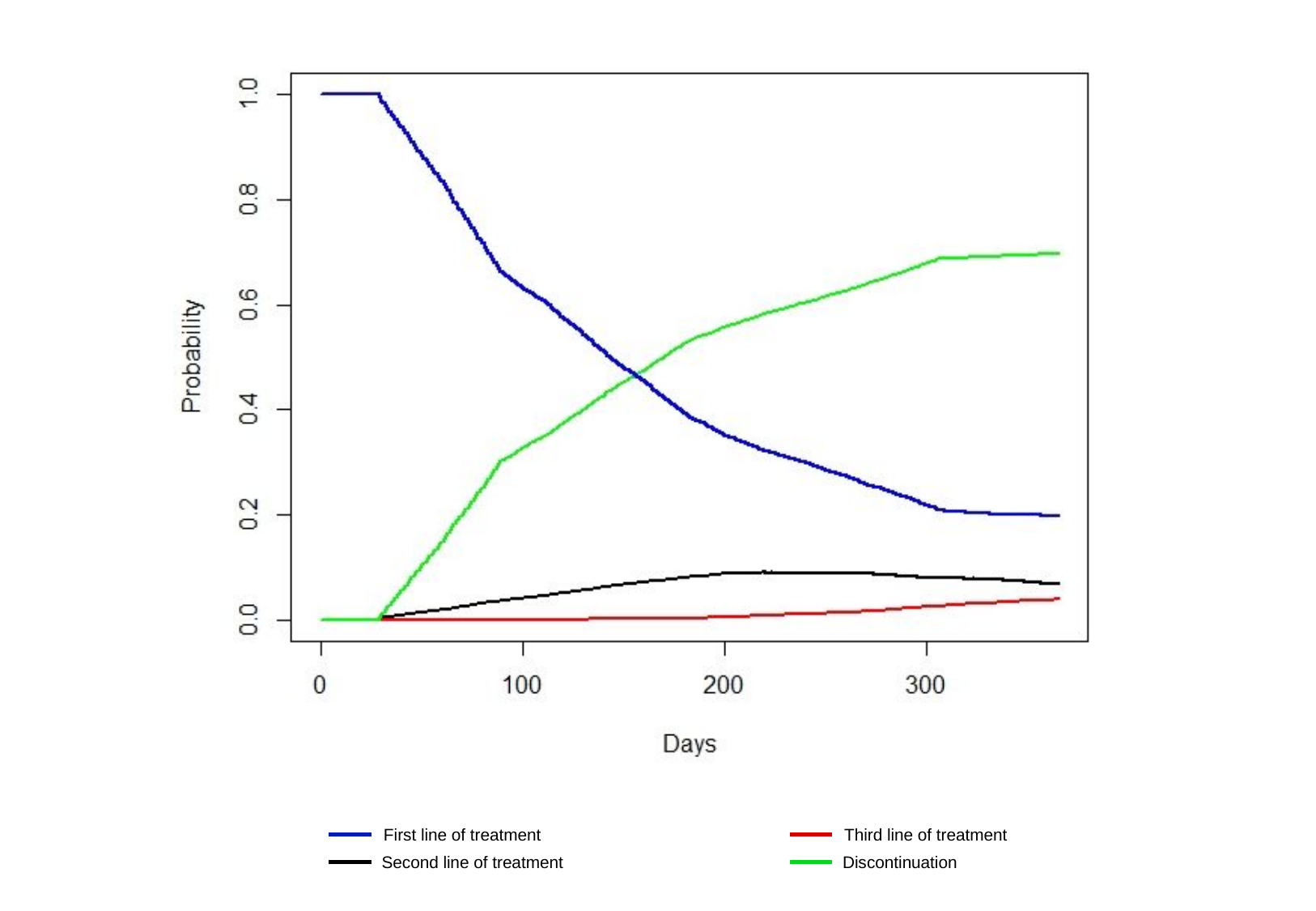

First line of treatment
Second line of treatment
Third line of treatment
Discontinuation
